# Assessment of causal effects of physical activity on the risk of osteoarthritis: a two-sample Mendelian randomization study

**DOI:** 10.1186/s12920-023-01681-x

**Published:** 2023-10-09

**Authors:** Bin Wang, Yang Liu, Yao-Chen Zhang, Zi-Yi Han, Jia-Lin Hou, Shuai Chen, Chuan Xiang

**Affiliations:** 1 Department of Orthopedic, Second Hospital of Shanxi Medical University, Taiyuan, 030001 China; 2 Department of Emergency Medicine, First Affiliated Hospital of Zhengzhou University, Zhengzhou, China; 3 Ministry of Education, Key Laboratory of Cellular Physiology at Shanxi Medical University, Taiyuan, China

**Keywords:** Mendelian randomization, Physical activity, Osteoarthritis, Causality

## Abstract

**Background:**

Growing evidence supports an association between physical activity (PA) and the risk of osteoarthritis (OA), but this may be influenced by confounding and reverse causality. Therefore, we performed a two-sample Mendelian randomization (MR) analysis to reveal the causal relationship between PA and OA.

**Methods:**

MR was performed to explore the causation of PA and OA with genetic variants as instrumental variables. The genetic variants were derived from the summary statistics of a large genome-wide association study meta-analysis based on the European population (*n* = 661,399), including self-reported leisure screen time (LST) and moderate-to-vigorous physical activity (MVPA), and Arthritis Research UK Osteoarthritis Genetics Consortium cohorts (417,596, 393,873 and 403,124 for overall, hip and knee OA, respectively). The major MR analysis used in this work was the inverse variance weighted (IVW) approach, and sensitivity, pleiotropy, and heterogeneity studies were performed to evaluate the validity of the findings.

**Results:**

IVW estimates indicated that LST had a risk effect on overall OA (odds ratio (OR) = 1.309, 95% confidence interval (CI): 1.198–1.430, *P* = 2.330 × 10^-9^), hip OA (OR = 1.132, 95% CI: 1.009–1.269, *P* = 0.034) and knee OA (OR = 1.435. 95% CI: 1.286–1.602, *P* = 1.225 × 10^-10^). In contrast, no causal relationship was found between MVPA and OA (overall OA: OR = 0.895, 95% CI: 0.664–1.205, *P* = 0.465; hip OA: OR = 1.189, 95% CI: 0.792–1.786, *P* = 0.404; knee OA: OR = 0.707, 95% CI: 0.490 -1.021, *P* = 0.064). In addition, we observed significant heterogeneity in instrumental variables, but no horizontal pleiotropy was detected.

**Conclusions:**

Recent findings demonstrated a protective impact of reducing LST on OA, independent of MVPA. This provides valuable insights into the role of physical activity in OA and offers lifestyle recommendations, such as reducing recreational sedentary behaviors and promoting appropriate exercise, for individuals at risk of OA.

**Supplementary Information:**

The online version contains supplementary material available at 10.1186/s12920-023-01681-x.

## Introduction

Osteoarthritis (OA) is a degenerative joint disease caused by various factors, such as obesity, aging, and strain, which leads to degenerative damage of articular cartilage and reactive hyperplasia of joint marginal and subchondral bone [1, 2]. Studies have shown that loss of complete meniscus function due to joint instability or abnormal mechanical load can cause OA in humans [3, 4]. Although our understanding of the disease has advanced considerably [5], there is still no cure for OA [6]. Therefore, it is critical to identify modifiable risk factors and seek potential treatments to slow the progression or even prevent the disease [7], of which physical activity (PA) is one. PA is movement done by skeletal muscles that demands energy expenditure beyond a baseline level and encompasses workouts, sports, and PA conducted in everyday life, work, transportation, and recreation [8].

PA is often seen as a factor that reduces the risk of OA. A meta-analysis based on data from 17 randomized clinical trials, including 1,705 patients, showed that resistance exercise was beneficial in reducing pain, relieving stiffness, and improving physical function in patients with OA of the knee [9]. One study concluded that higher levels of PA were associated with the maintenance of physical function and encouraged PA in older adults at risk of OA [10]. However, one randomized controlled trial showed that high-intensity PA did not result in improvements in physical functioning in patients with knee OA compared to low-intensity PA [11], and another study came to a similar conclusion [12]. Additionally, a study found a significant association between reduced sedentary behavior and a subsequent decrease in functional decline in the future, independent of time spent in MVPA [13], another study reached a similar conclusion [14]. In contrast, studies have shown that non-pharmacological OA prevention is not only based on regular PA but also on reducing the amount of time spent in sedentary activities throughout the day and changing sedentary behaviors [15]. However, there is evidence that in the general adult individuals, self-reported diagnosis of knee/hip OA was not associated with low, moderate or high levels of PA [16]. Because of the inherent limitations of reverse causality and potential bias in observational studies, the role of PA in OA etiology has yet to be convincingly concluded to date.

Mendelian randomization (MR) may be used for causal inference in epidemiology [17], which employs genetic variations closely associated with exposure as the exposure device to infer the causal influence of exposure on the result from non-experimental data. Due to the fact that genetic variations are allocated randomly at the time of conception, genetic analysis technologies provide substantial benefits by removing potential sources of error and establishing a clear chain of causation. In MR Analysis, the hypothesis of a causal relationship between exposure and outcome is supported if the observed genetic variation associated with exposure is also associated with the risk of the outcome. In addition, coupled with the wide availability of published genetic associations, MR became a time and cost-effective method and contributed to its growing popularity in evaluating and screening potential causal associations [18].

In this study, we performed a two-sample MR analysis, which allows the selection of genetic variants as instruments for a risk factor (PA) in one sample and explore associations of these variants with outcomes (OA) in another sample [19, 20]. By overcoming the requirement for estimating the exposure and outcomes in the same dataset, this approach is able to explore associations in publicly available aggregated statistics from large GWAS and increase power accordingly.

## Methods

### Data sources

Summary statistics for PA were derived from a large genome-wide association study (GWAS) meta-analysis among 661,399 participants of European ancestry [21]. Two PA phenotypes were analyzed in this study, including self-reported leisure screen time (LST) and moderate-to-vigorous physical activity (MVPA). Activities such as swimming and jogging were classified as MVPA, while activities such as watching television, playing video games, and sitting at the computer were classified as LST. Different studies used different questionnaires to assess PA, so population-specific characteristics were defined to make optimal use of the available data. GWAS for the PA trait used an additive genetic model that was adjusted for age, age-squared, and other study-specific covariates. The standard quality control checks were conducted for GWAS data before imputation.

Summary statistics for overall OA, hip OA, and knee OA were obtained from Arthritis Research UK Osteoarthritis Genetics Consortium cohorts, which were based on 417,596 (39,427 OA cases and 378,169 controls), 393,873 (15,704 OA cases and 378,169 controls), 403,124 (24,955 OA cases and 378,169 controls) European individuals [22]. The potential confounders associated with OA, including smoking, body mass index (BMI) [23], and drinking [24] were adjusted.

### Selection of instrumental variables

**Fig. 1:**
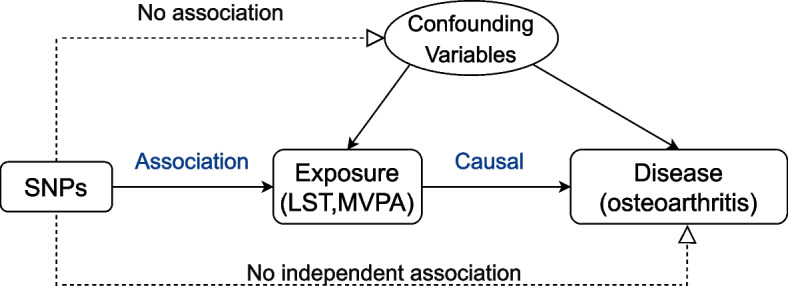
Schematic diagram of Mendelian randomization study. This design is under the hypothesis that genetic instrumental variables are associated with exposure, but not with confounders, and the genetic instrumental variable affect outcome only through exposure. Genetic instrumental variables represent single nucleotide polymorphisms

In the two-sample MR analysis of the present study, the MR approach we used must meet the following three assumptions [25]: (a) genetic variants must be closely linked to the exposure (PA); (b) genetic variants wouldn’t be influenced by other factors; (c) genetic variants must only be related to the risk of outcomes (OA) through exposures (PA), not through other pathways. Schematic diagram of Mendelian randomization study is shown in Fig. 1.

SNPs (*P* < 5 × 10^-8^) associated with a corresponding exposure at the genome-wide significance threshold were extracted in our current study. To ensure independence among each selected SNP constituting each genetic instrument, a Linkage disequilibrium analysis of the corresponding SNPs for each instrumental variable (r2 = 0.001 and kb = 10,000 kb) was applied. Then, the PhenoScanncer GWAS database was used to assess and delete SNPs correlated to potential confounding factors (including smoking, drinking, and BMI) and outcomes.

### MR analysis

**Fig. 2:**
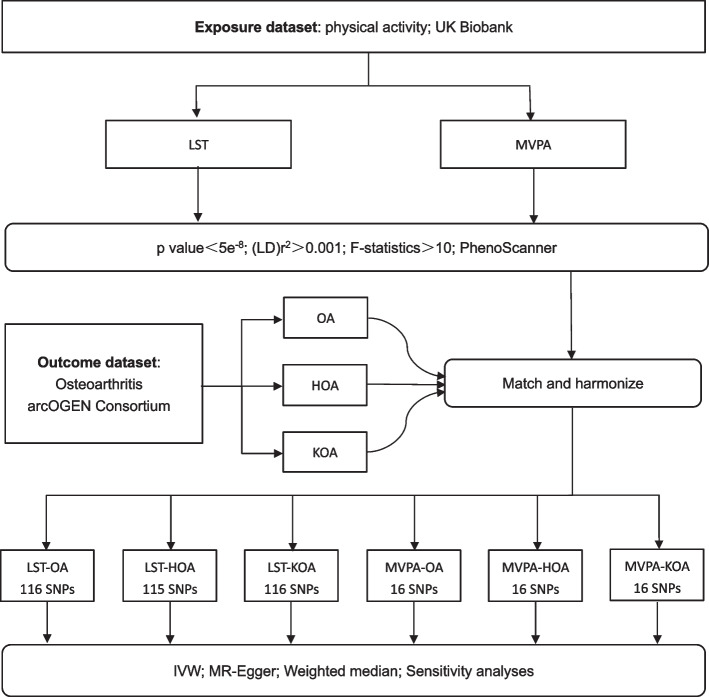
Schematic diagram of a bidirectional MR study of LST, MVPA and OA. Abbreviations: LST, leisure screen time. MVPA, moderate to vigorous intense physical activity during leisure screen time. OA, osteoarthritis. LD, linkage disequilibrium. KOA, knee osteoarthritis. HOA, hip osteoarthritis. SNPs, single-nucleotide polymorphisms. MR, Mendelian randomization. IVW, inverse variance weighted

The robust inverse-variance weighted (IVW) regression was adopted as the main analysis method [26], while the robust MR-Egger and weighted median methods were applied as a complement to IVW. IVW is a popular technique for MR analysis, which obtains ratio estimates by dividing SNP-outcome effects by SNP-exposure effects, where the intercept is restricted to zero. The weighted median approach was utilized to provide reliable estimates, in which the MR analysis retained its robustness when < 50% of genetic variation was invalid [27]. All analyses were completed with the help of the “TwoSampleMR” package in the R software environment (version 4.1.2). Our flow chart is shown in Fig. 2.

### Sensitivity analysis

A leave-one-out sensitivity analysis was performed to test if one single SNP was responsible for a disproportionate amount of variance in the relationship [28, 29]. The MR analysis was carried out again, leaving out each SNP in turn. Subsequently, a comprehensive analysis of all SNPs was run and shown for evaluation [30]. The MR result was robust if the difference was not significant.

### Pleiotropy assessment

The MR-Egger regression is an efficient method for assessing horizontal pleiotropy. It establishes a weighted linear regression between the result coefficients and the exposure coefficients, with an intercept term indicating the average pleiotropic impact. If the intercept estimate of the MR-Egger regression significantly deviated from 0 (*P* value for MR-Egger intercept < 0.05), the existence of horizontal pleiotropic was considered [31] and corrected by outlier removal (MR-PRESSO outlier test).

### Heterogeneity assessment

The Cochran’s Q statistic was employed to assess heterogeneity between SNPs, and a *p*-value < 0.1 would be considered significant heterogeneity [32]. Moreover, the scatter plots were used to visually evaluate the heterogeneity of SNPs.

## Results

### LST and OA

#### The character of selected SNPs

We identified 115 independent SNPs associated with LST with a *p*-value of less than 5 × 10^–8^. All SNPs were independent in the linkage disequilibrium clusters. By searching the PhenoScanner database, we removed SNPs related to previously reported confounders. In addition, there were 116, 115, and 116 SNPs for overall OA, hip OA, and knee OA, respectively. Six palindromic genetic variants (rs10759934, rs10889189, rs13380133, rs1736523 rs6130716, and rs843090) and one SNP for an incompatible allele (rs9889540) were removed from these SNPs, respectively. As a result, 108 SNPs were eventually included in the MR and sensitivity analyses. Detailed information on these SNPs is displayed in Supplemental Tables 1, 2 and 3.

#### MR analysis

**Fig. 3:**
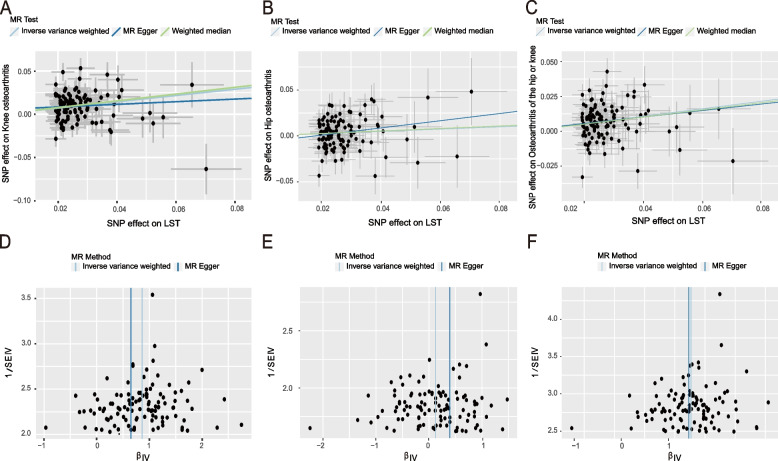
Scatterplot, funnel plot and leave-one-out plot of LST on OA causal effects. **A**-**C** Scatterplot for IVW, MR-Egger and WM analysis methods demonstrating the effect of LST on knee OA (**A**), hip OA (**B**), and overall OA (**C**); **D**-**F** Funnel plot of LST on knee OA (**D**), hip OA (**E**), and overall OA (**F**) to suggest evidence of substantial heterogeneity. Abbreviations: LST, leisure screen time. OA, osteoarthritis. SNPs, single-nucleotide polymorphisms. MR, Mendelian randomization. IVW, inverse variance weighted. WM, weighted median

Inverse variance weighted estimates indicated that leisure screen time had a risk effect on overall OA (odds ratio [OR] = 1.309, 95% confidence interval [CI]: 1.198–1.430, *P* = 2.330 × 10^-9^), hip OA (OR = 1.132, 95% CI: 1.009–1.269, *P* = 0.034), and knee OA (OR = 1.435, 95% CI: 1.286–1.602, *P* = 1.225 × 10^-10^). Analyses using the MR-Egger and weighted median methods yielded similar effect estimates. Heterogeneity was noted between individual SNPs for leisure screen time and OA in the heterogeneity analysis (*p* < 0.05). No lateral pleiotropy was identified in the Egger intercept test (overall OA: intercept = 0.001, *P* = 0.849; hip OA: intercept = -0.007, *P* = 0.286; knee OA: intercept = 0.005, *P* = 0.386) (Table 1). The “leave-one-out” test indicated no outliers in these SNPs. Scatterplot and funnel plot are shown in (Fig. 3A-F).

**Table 1:** The causal effect of LST on overall OA, hip OA and knee OA

Exposure	Outcome	SNPs	MR methods	OR	95% CI	Q_pval	Intercept_pval
LST	overall OA	108	IVW	1.309	(1.198, 1.430)	8.944E-07	0.849
			MR- Egger	1.261	(0.852, 1.867)	6.663E-07	
			WM	1.292	(1.167, 1.430)		
	hip OA	108	IVW	1.132	(1.009, 1.269)	0.025	0.286
			MR- Egger	1.482	(0.894, 2.456)	0.027	
			WM	1.146	(0.985, 1.332)		
	knee OA	108	IVW	1.435	(1.286, 1.602)	9.241E-08	0..386
			MR- Egger	1.163	(0.715, 1.891)	9.412E-08	
			WM	1.476	(1.306, 1.667)		

The Q_pval was executed by the MR-Egger and IVW methods to detect heterogeneity. The intercept *p*-value was derived from the pleiotropy test of MR-Egger regression

*Abbreviations*: *LST* leisure screen time, *OA* osteoarthritis, *SNPs* single-nucleotide polymorphisms, *MR* Mendelian randomization, *CI* confidence interval, *OR* odds ratio, *IVW* inverse variance-weighted, *WM* weighted median

### MVPA and OA

#### The character of selected SNPs

We identified 16 independent SNPs related to MVPA with a *p*-value of less than 5 × 10^-8^. All SNPs were independent in the LD clustering. Through a search of the PhenoScanner database, we removed SNPs associated with previously reported confounders. In addition, the SNPs used for overall OA, hip OA, and knee OA were all 16. One palindromic genetic variant (rs11130222) was removed from these SNPs. Therefore, the final number of SNPs included in MR and sensitivity analyses was 15. Detailed information on these SNPs is listed in Supplemental Tables 4, 5 and 6.

#### MR analysis

**Fig. 4:**
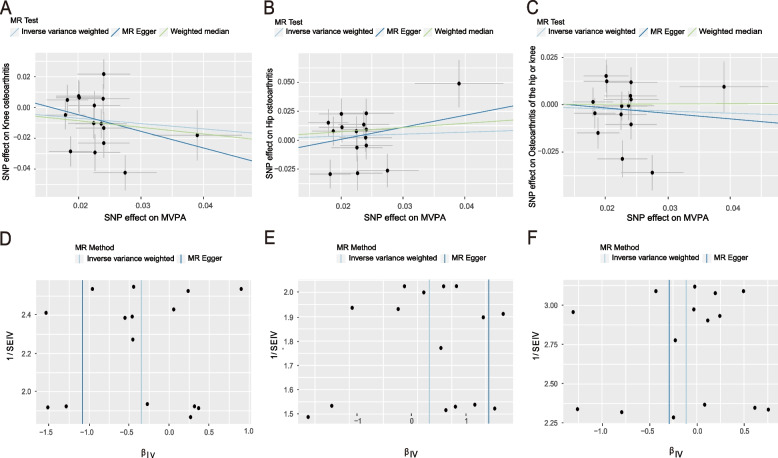
Scatterplot, funnel plot and leave-one-out plot of MVPA on OA causal effects. **A**-**C** Scatterplot for IVW, MR-Egger and WM analysis methods demonstrating the effect of MVPA on knee OA (**A**), hip OA (**B**), and overall OA (**C**); **D**-**F** Funnel plot of MVPA on knee OA (**D**), hip OA (**E**), and overall OA (**F**) to suggest evidence of substantial heterogeneity. Abbreviations: MVPA, moderate to vigorous intense physical activity during leisure screen time. OA, osteoarthritis. SNPs, single-nucleotide polymorphisms. MR, Mendelian randomization. IVW, inverse variance weighted. WM, weighted median

It was obvious that regardless of the instrumental SNP threshold, the use of inverse variance weighting methods demonstrated no significant evidence for a causal relationship between performing MVPA and OA (overall OA: OR = 0.895, 95% CI: 0.664–1.205, *P* = 0.465; hip OA: OR = 1.189, 95% CI: 0.792–1.786, *P* = 0.404; knee OA: OR = 0.707, 95% CI: 0.490–1.021, *P* = 0.064. Besides, the results of the study using weighted medians and MR-Egger are consistent with those of IVW. Heterogeneity was found between individual SNPs and OA for MVPA in the heterogeneity analysis (*P* < 0.05). No horizontal pleiotropy was witnessed following the Egger intercept test (All *p*-values of MR-Egger regression > 0.05) (Table 2). The “leave-one-out” test showed no outliers in these SNPs. Scatterplot and funnel plot are shown in (Fig. 4A-F).

**Table 2:** The causal effect of MVPA on overall OA, hip OA and knee OA

Exposure	Outcome	SNPs	MR methods	OR	95%CI	Q_pval	Intercept_pval
MVPA	overall OA	15	IVW	0.895	(0.664, 1.205)	8.365E-04	0.854
			MR- Egger	0.748	(0.112, 4.992)	4.890E-04	
			WM	0.014	(0.764, 1.345)		
	hip OA	15	IVW	1.189	(0.792, 1.786)	0.011	0.510
			MR- Egger	2.837	(0.222, 36.277)	0.009	
			WM	1.440	(0.933, 2.223)		
	knee OA	15	IVW	0.707	(0.490, 1.021)	6.427E-04	0.538
			MR- Egger	0.338	(0.033, 3.426)	5.368E-04	
			WM	0.651	(0.455, 0.932)		

The Q_pval was executed by the MR-Egger and IVW methods to detect heterogeneity

The intercept *p*-value was derived from the pleiotropy test of MR-Egger regression

*Abbreviations*: *MVPA* moderate to vigorous intense physical activity during leisure screen time, *OA* osteoarthritis, *SNPs* single-nucleotide polymorphisms, *MR* Mendelian randomization, *CI* confidence interval, *OR* odds ratio, *IVW* inverse variance-weighted, *WM* weighted median

## Discussion

This study is the first MR analysis on the causal relationship between PA and OA. Based on the data shown in the results, we found that reducing LST had a protective effect against OA, which is largely consistent with the findings of most prevenient observational studies [33–35]. Although its odds ratio (OR) value is relatively small, which means the effect of LST on OA is relatively small, it still provides a promising approach for preventing OA.

A large number of observational studies have documented an association between PA and OA. Among patients with knee or hip OA, regular PA is beneficial in maintaining physical function and reducing pain symptoms. Specifically, a cohort study with 12,796 participants has demonstrated that sustained long-term pain relief is confirmed with supervised exercise therapy in knee OA patients, regardless of the initial level of PA [36]. Notably, persistent physical inactivity and sedentary behavior are generally associated with the developing risk of functional limitation in adults with knee OA independent of sedentary category [33], with a similar result also found in another study [35]. However, some studies have come to the opposite conclusion. Sixty-nine studies from 23 countries have shown that multiple activities, including lifting weights and whole-body vibration, increase the risk of developing OA in men and women [37]. Besides, including 6 global, community-based cohort studies, an international meta-analysis of individual participant-level data has suggested that physiologic energy expenditure during recreational activities and time spent in PA are not related to the risk of OA. Compared with large-scale prospective clinical trials requiring long-term observation, the MR analysis offers an efficient way to explore the causality between PA and the risk of OA [38]. Using the MR approach, our study confirmed that reducing LST would have a beneficial effect on OA. Therefore, the incidence of OA can be reduced by promoting lifestyle management, such as reducing recreational sedentary activities and encouraging appropriate exercise.

One possible explanation is that PA could function by inducing the expression and release of irisin in muscle tissue [39, 40]. On the one hand, the irisin-integrin-wnt/β-catenin-Runx2 regulatory axis may form a signaling axis [41–43], regulating the differentiation and proliferation of osteoblasts. On the other hand, irisin can inhibit the apoptosis of chondrocytes by reducing inflammatory factors and matrix metalloproteinases in chondrocytes [44, 45], thereby exerting a protective effect on OA [46]. Besides, PA can function as an underlying immunomodulatory therapy. PA leads in dramatically increased T-regulatory cells, reduced immunoglobulin production, and alterations in the Th1/Th2 ratio, and has been proven to attenuate inflammatory events in patients and animal models of inflammatory disorders [47, 48]. As recently reported, the considerable improvement in function and pain following physical exercise intervention in overweight adults with knee OA is partially modulated by alterations in a mix of inflammatory markers, including IL-6, TNF-α, IL-1sR, and CRP [49]. These observational studies implied that PA might affect the pathogenesis of OA through modulation of inflammation.

The current study had several main advantages. Firstly, by assigning randomly allocated alleles to offspring and essentially immobilizing them at conception, MR results are less susceptible to confounding and reverse causal measurement errors than traditional observational studies. Secondly, our study utilized large-scale GWAS datasets from two distinct European populations to reduce the effects of population stratification and increase statistical power for identifying a causal inference. Thirdly, multiple sensitivity analyses revealed no pleiotropy, further confirming the robustness of our results.

Nonetheless, there were several limitations worth mentioning in our study. Firstly, since the PA was ascertained by self-report, this was prone to recall and response bias. Secondly, given that our findings were mainly limited to participants of European ancestry, our conclusions may not be suitable for generalization to other kinds of populations. Thirdly, our MR analysis only yielded genetic evidence. Further larger or multi-ethnicity studies or prospective clinical trials should be conducted to validate our findings in the future, explore the underlying mechanisms of this possible causality and further explore related mechanisms. Lastly, our data had not demonstrated a causal relationship between MVPA and OA, so we should interpret the results cautiously.

## Conclusions

Our two-sample MR study suggested reducing LST could reduce the risk of OA, independently of MVPA. This research has the potential to shed new light on the association between PA and OA risk and to suggest potential lifestyle adjustments, such as reducing recreational sedentary activities and encouraging proper exercise, for those who are predisposed to developing OA.

### Supplementary Information

**Additional file 1: Supplemental Table 1.** Summary of the 108 SNPs associated with LST and knee OA. LST, leisure screen time; OA, Osteoarthritis. **Supplemental Table 2.** Summary of the 108 SNPs associated with LST and hip OA. LST, leisure screen time; OA, Osteoarthritis. **Supplemental Table 3.** Summary of the 108 SNPs associated with LST and overall OA. LST, leisure screen time; OA, Osteoarthritis. **Supplemental Table 4.** Summary of the 15 SNPs associated with MVPA and knee OA. MVPA, moderate-to-vigorous physical activity; OA, Osteoarthritis. **Supplemental Table 5.** Summary of the 15 SNPs associated with MVPA and hip OA. MVPA, moderate-to-vigorous physical activity; OA, Osteoarthritis. **Supplemental Table 6.** Summary of the 15 SNPs associated with MVPA and overall OA. MVPA, moderate-to-vigorous physical activity; OA, Osteoarthritis.

## Data Availability

GWAS summary statistics about physical activity are available in the GWAS Catalog (https://www.ebi.ac.uk/gwas/downloads/summary-statistics) using accession numbers GCST90104341 and GCST90104339. And we also could download GWAS summary statistics of overall OA, hip OA, and knee OA from the publicly available IEU GWAS database (https://gwas.mrcieu.ac.uk/datasets/ebi-a-GCST007092/, https://gwas.mrcieu.ac.uk/datasets/ebi-a-GCST007091/, https://gwas.mrcieu.ac.uk/datasets/ebi-a-GCST007090/).
