# Supplementary material for: Assessment of causal effects of physical activity on the risk of osteoarthritis: a two-sample Mendelian randomization study
Source: BMC Med Genomics. 2023 Oct 9;16:237. doi: 10.1186/s12920-023-01681-x (PMC10561455; doi:10.1186/s12920-023-01681-x)
Supplement: Supplementary file 1 — Additional file 1: Supplemental Table 1. Summary of the 108 SNPs associated with LST and knee OA. LST, leisure screen time; OA, Osteoarthritis. Supplemental Table 2. Summary of the 108 SNPs associated with LST and hip OA. LST, leisure screen time; OA, Osteoarthritis. Supplemental Table 3. Summary of the 108 SNPs associated with LST and overall OA. LST, leisure screen time; OA, Osteoarthritis. Supplemental Table 4. Summary of the 15 SNPs associated with MVPA and knee OA. MVPA, moderate-to-vigorous physical activity; OA, Osteoarthritis. Supplemental Table 5. Summary of the 15 SNPs associated with MVPA and hip OA. MVPA, moderate-to-vigorous physical activity; OA, Osteoarthritis. Supplemental Table 6. Summary of the 15 SNPs associated with MVPA and overall OA. MVPA, moderate-to-vigorous physical activity; OA, Osteoarthritis. [file 12920_2023_1681_MOESM1_ESM.pdf]

Supplementary Material

Supplementary Tables

Supplemental Table 1: Summary of the 108 SNPs associated with LST and knee OA. LST, leisure screen time; OA, Osteoarthritis

SNP	chromosome	Position	Effect allele	Other allele	Effect allele frequency	Effect on LSP			Effect on knee OA		
						Beta	SE	P value	Beta	SE	P value
rs10044672	5	138290479	C	G	0.6518	-0.0233	0.0037	1.79E-10	-0.0165	0.0123	0.1783
rs10159545	10	21766969	C	G	0.6504	-0.0347	0.0041	9.62E-18	-0.0336	0.0123	0.00627697
rs10206568	2	217250099	C	G	0.71	0.021	0.0038	4.41E-08	0.0205	0.0129	0.1107
rs10222987	4	185946130	A	G	0.6355	-0.0209	0.0036	9.02E-09	0.0121	0.0121	0.319
rs10273241	7	8085405	A	G	0.4385	-0.0192	0.0035	4.49E-08	0.0126	0.0118	0.2835

rs10400776	14	97326366	A	C	0.2593	-0.0259	0.0044	3.45E-09	0.002	0.0133	0.8809
rs10427614	22	29928932	A	G	0.7708	-0.0249	0.0046	4.94E-08	-0.0214	0.0139	0.1224
rs10432338	2	147806785	T	G	0.1823	0.0331	0.005	2.61E-11	0.0235	0.0151	0.1203
rs10792966	11	71544105	C	G	0.6876	0.0237	0.0041	1.06E-08	0.0204	0.0126	0.1052
rs11066368	12	109790342	A	G	0.3874	0.021	0.0036	4.31E-09	0.0046	0.012	0.704399
rs11074655	16	10300931	T	C	0.5879	-0.0205	0.0035	6.51E-09	0.0039	0.0118	0.744201
rs113367286	7	140144414	T	C	0.2787	-0.0251	0.0043	4.99E-09	-0.0057	0.013	0.6598
rs11597584	10	66401600	T	C	0.3432	-0.0224	0.0041	4.15E-08	-1.00E-04	0.0124	0.9905
rs117737827	15	63804248	T	C	0.1558	-0.0299	0.0053	1.69E-08	-0.0259	0.0161	0.1076
rs1188840	6	139214819	A	G	0.6509	0.0236	0.004	4.40E-09	-0.0153	0.0122	0.2112
rs12188899	5	59670706	A	G	0.797	-0.0274	0.0048	1.09E-08	0.0125	0.0146	0.3916

rs1219798	10	125400180	T	C	0.1746	0.0269	0.0046	4.64E-09	-0.0259	0.0154	0.092221
rs12206846	6	108238917	A	G	0.4054	0.0199	0.0036	2.10E-08	0.0027	0.0119	0.818
rs12214364	6	67556372	T	G	0.5869	-0.0197	0.0036	4.87E-08	-0.0021	0.012	0.8611
rs12469397	2	193762132	A	G	0.4922	-0.0258	0.0038	2.07E-11	-0.0149	0.0117	0.2015
rs12531466	7	98933552	T	C	0.8738	0.034	0.0061	1.91E-08	-0.0058	0.0175	0.7415
rs12707100	7	133274376	T	C	0.7984	-0.0265	0.0048	2.84E-08	0.0045	0.0145	0.7556
rs127204	1	112277693	A	C	0.5998	-0.0258	0.0039	3.59E-11	-0.0119	0.0119	0.3172
rs12992995	2	175197545	A	C	0.2755	-0.0258	0.0043	2.10E-09	-1.00E-04	0.0131	0.9969
rs13011181	2	100732446	T	C	0.7615	0.0278	0.0045	7.34E-10	-0.0138	0.0137	0.3142
rs13089152	3	84765574	T	C	0.6691	-0.0234	0.0041	1.53E-08	0.0018	0.0126	0.8873
rs13144223	4	17872707	T	C	0.8683	-0.0293	0.0051	1.19E-08	-0.0116	0.0172	0.4996
rs13168806	5	120071604	A	C	0.3752	0.0225	0.004	1.52E-08	-0.0031	0.012	0.7984

rs138225564	3	83063804	A	G	0.6479	0.0251	0.0046	4.48E-08	0.0099	0.0144	0.4907
rs1391954	11	88575965	T	G	0.4439	0.0245	0.004	1.51E-09	-0.013	0.0117	0.2686
rs144519232	3	48866313	C	G	0.9112	-0.0404	0.0068	3.00E-09	-0.021	0.0206	0.3103
rs1483796	7	49871114	A	G	0.3988	-0.0199	0.0036	2.15E-08	-0.0043	0.0119	0.7175
rs151431	4	103128298	A	G	0.2364	0.0277	0.0048	7.14E-09	0.0326	0.015	0.0296203
rs17801257	20	58892520	A	G	0.1247	-0.0292	0.0053	2.92E-08	0.0059	0.0178	0.7422
rs1837687	2	191238799	C	G	0.609	0.0222	0.0039	1.85E-08	0.0075	0.012	0.5295
rs1938450	1	68359482	A	G	0.3312	-0.0226	0.0041	2.66E-08	-0.0085	0.0124	0.4925
rs1968557	5	112029044	T	C	0.4961	-0.0193	0.0035	3.92E-08	-0.017	0.0117	0.1461
rs2344118	8	30788634	A	C	0.3611	0.0208	0.0037	2.65E-08	-0.0021	0.0124	0.8651
rs2417261	12	13414139	T	G	0.1085	0.0328	0.0056	5.88E-09	0.0099	0.0189	0.6011

rs243077	2	60616682	A	G	0.4197	0.0242	0.0039	5.26E-10	-0.0172	0.0118	0.146
rs2452681	11	65814842	A	G	0.7595	-0.0289	0.0045	1.41E-10	0.0104	0.0137	0.4465
rs2473977	6	113454213	A	T	0.3862	0.0195	0.0036	4.60E-08	0.0102	0.012	0.3946
rs27694	5	96164109	A	T	0.2087	-0.0236	0.0043	3.66E-08	-0.0328	0.0144	0.0226898
rs28808010	9	22492584	T	C	0.949	-0.0488	0.0089	4.41E-08	0.0038	0.0275	0.8889
rs2965216	19	13071082	A	G	0.4549	0.0212	0.0039	4.61E-08	-0.0134	0.0118	0.2563
rs34468927	19	37400719	A	G	0.8552	0.0301	0.0055	4.00E-08	0.0101	0.018	0.574101
rs34517439	1	78450517	A	C	0.1232	0.0368	0.0059	4.36E-10	-0.007	0.0179	0.6955
rs34898652	1	43974091	A	G	0.3825	-0.0228	0.0039	6.54E-09	-0.0337	0.012	0.00502597
rs349368	8	73427898	T	C	0.081	0.0349	0.0064	4.73E-08	0.0176	0.0215	0.4115
rs35041900	1	171451621	T	C	0.0932	0.0389	0.0066	3.77E-09	0.0122	0.0201	0.5454
rs362307	4	3241845	T	C	0.0739	0.0417	0.0075	2.64E-08	-0.0232	0.0222	0.2942

rs3781412	10	126715154	A	G	0.6005	-0.0208	0.0036	8.38E-09	-0.0122	0.0119	0.3036
rs3853705	2	44261892	T	C	0.6858	0.0228	0.004	1.43E-08	0.0066	0.0126	0.6016
rs4275659	12	123447928	T	C	0.2838	-0.0217	0.0039	1.99E-08	0.0036	0.013	0.782699
rs4311996	10	103735978	A	T	0.3873	-0.0217	0.0039	3.56E-08	0.0101	0.012	0.4007
rs4326129	5	151996816	A	T	0.6807	0.0226	0.0041	3.86E-08	-0.0032	0.0125	0.796
rs4375301	1	98298371	A	G	0.7673	-0.0265	0.0045	5.08E-09	-0.0173	0.0138	0.2113
rs4460001	4	130275243	A	C	0.4324	-0.0193	0.0035	4.45E-08	-0.0135	0.0119	0.2552
rs4599438	4	77028142	A	G	0.3867	0.0212	0.0038	3.54E-08	-0.0085	0.012	0.478
rs4615256	5	62700867	A	G	0.5275	-0.0205	0.0035	3.33E-09	-0.0176	0.0117	0.1321
rs4680609	3	165598022	A	G	0.6051	-0.0221	0.0039	1.89E-08	-0.0161	0.0129	0.2137
rs474252	11	95619292	C	G	0.3909	-0.0218	0.0038	1.29E-08	0.0133	0.0119	0.2664
rs4948288	10	63584533	A	G	0.4132	-0.0195	0.0035	3.31E-08	-0.0104	0.0118	0.3809

rs55716899	3	71498561	T	C	0.5712	0.0217	0.0039	2.24E-08	0.0075	0.0118	0.5225
rs566017137	2	157149797	C	G	0.9639	-0.0656	0.0108	1.23E-09	0.0223	0.0339	0.51
rs572994	6	12686856	T	C	0.3854	-0.0242	0.0039	8.36E-10	-0.0043	0.012	0.720299
rs58087899	1	1863026	A	G	0.4556	-0.0222	0.004	4.35E-08	-0.0241	0.0127	0.0573099
rs6010651	20	62418243	A	C	0.6201	0.0235	0.004	3.34E-09	0.0014	0.012	0.9047
rs60344242	3	70472539	T	C	0.1922	0.0271	0.0049	2.48E-08	-0.0043	0.0148	0.7732
rs6102913	20	41202958	T	C	0.4736	-0.0193	0.0035	2.97E-08	0.0038	0.0116	0.7467
rs61166637	4	140771814	C	G	0.6711	0.0242	0.0043	1.48E-08	-0.0079	0.0124	0.5213
rs61813324	1	156049877	T	C	0.1361	0.031	0.0057	4.37E-08	0.0037	0.0172	0.8287
rs62009152	15	78001011	T	C	0.7423	0.0259	0.0044	3.63E-09	0.0015	0.0133	0.9091
rs62057680	16	29986627	T	C	0.9204	-0.0408	0.0072	1.56E-08	-0.0051	0.0218	0.8155
rs62068672	16	89647015	T	C	0.798	0.0263	0.0048	4.09E-08	0.024	0.0157	0.1267

rs62129987	19	10744807	T	C	0.6259	-0.0274	0.004	5.68E-12	-0.0158	0.0131	0.2278
rs62134192	2	45053345	C	G	0.9545	0.0524	0.0093	1.68E-08	-0.0291	0.0281	0.3013
rs62275609	3	90356910	A	G	0.2509	0.0248	0.0044	2.29E-08	-0.0271	0.0146	0.0636605
rs6430085	2	146255869	T	G	0.4936	-0.0222	0.0039	8.66E-09	-0.0082	0.0117	0.484
rs657412	13	99047250	T	G	0.1047	-0.033	0.0058	1.40E-08	0.0164	0.0191	0.3906
rs66723933	5	62360995	A	C	0.1743	-0.028	0.0051	3.23E-08	-0.0091	0.0154	0.5571
rs6674314	1	243920895	A	G	0.8134	0.0274	0.0049	2.30E-08	-0.0058	0.0149	0.696
rs67369356	5	77323022	T	C	0.2451	-0.0244	0.0045	4.23E-08	-0.0158	0.0135	0.2423
rs6741157	2	104421850	T	C	0.4526	0.0223	0.0039	8.69E-09	0.012	0.0117	0.3072
rs6743939	2	215254537	T	G	0.5831	0.0203	0.0035	9.03E-09	-0.0273	0.0118	0.0209802
rs6763786	3	74966094	A	G	0.2296	0.0228	0.0041	3.30E-08	0.0018	0.0138	0.8976
rs67943625	3	50795628	A	G	0.9383	-0.0558	0.0101	3.02E-08	-0.0418	0.0316	0.1859

rs6857	19	45392254	T	C	0.1689	-0.0369	0.0047	5.80E-15	-0.0399	0.0155	0.01011
rs71639293	5	92995013	A	G	0.7974	0.0266	0.0048	2.80E-08	-0.0095	0.0145	0.5147
rs7227386	18	41594173	A	C	0.6831	-0.023	0.0041	2.48E-08	0.0097	0.0125	0.4372
rs73028181	6	166115539	T	G	0.0262	0.0704	0.012	4.40E-09	0.0482	0.0364	0.1847
rs73051647	12	6835160	A	G	0.0964	0.0377	0.0065	7.28E-09	0.0377	0.0197	0.05633
rs73420302	17	77768068	C	G	0.1785	-0.03	0.0051	3.04E-09	-0.0057	0.0153	0.7076
rs73697465	7	53900433	A	C	0.1662	0.0294	0.0052	1.39E-08	0.0266	0.0158	0.0909599
rs74996610	12	24075007	C	G	0.9545	-0.0513	0.0093	3.12E-08	-0.0098	0.0278	0.724499
rs7532726	1	209760936	A	C	0.7355	0.0218	0.0039	2.40E-08	-0.0173	0.0132	0.1906
rs7579662	2	161915810	A	G	0.393	0.0216	0.0039	4.15E-08	-0.019	0.012	0.1119
rs7803571	7	110993511	T	C	0.3722	0.0197	0.0036	4.74E-08	0.0124	0.0121	0.3025
rs78394231	6	107649123	T	C	0.9015	-0.0381	0.0065	3.53E-09	0.0436	0.0197	0.0265003

rs7843666	8	10742059	A	G	0.4938	0.0193	0.0035	3.57E-08	-0.0433	0.0117	0.000205902
rs7875078	9	14494845	A	C	0.4517	0.0204	0.0035	7.41E-09	-0.0039	0.0117	0.738901
rs9277976	6	33292745	T	C	0.9053	-0.0391	0.0069	1.41E-08	-0.0105	0.0199	0.5995
rs9411327	9	134794501	A	G	0.1654	0.03	0.0052	7.59E-09	0.0038	0.0157	0.8064
rs948537	18	35125161	T	C	0.2281	0.0277	0.0046	1.83E-09	-0.0179	0.014	0.2
rs9557370	13	100704249	A	G	0.2562	0.0301	0.0044	9.66E-12	3.00E-04	0.0134	0.9849
rs9867121	3	114631548	A	C	0.1829	-0.0317	0.005	2.02E-10	0.0032	0.0151	0.8324
rs9889540	17	60789059	C	G	0.3863	0.0234	0.0041	1.78E-08	0.005	0.012	0.680299
rs993884	5	7378895	T	C	0.6328	-0.0218	0.004	4.55E-08	-0.0049	0.0121	0.6851

Supplemental Table 2: Summary of the 108 SNPs associated with LST and hip OA. LST, leisure screen time; OA, Osteoarthritis.

SNP	chromosome	Position	Effect allele	Other allele	Effect allele frequency	Effect on LSP			Effect on hip OA		
						Beta	SE	P val1ue	Beta	SE	P value
rs10044672	5	138290479	C	G	0.6518	-0.0233	0.0037	1.79E-10	-0.0165	0.0123	0.1783
rs10159545	10	21766969	C	G	0.6504	-0.0347	0.0041	9.62E-18	-0.0336	0.0123	0.00627697
rs10206568	2	217250099	C	G	0.71	0.021	0.0038	4.41E-08	0.0205	0.0129	0.1107
rs10222987	4	185946130	A	G	0.6355	-0.0209	0.0036	9.02E-09	0.0121	0.0121	0.319
rs10273241	7	8085405	A	G	0.4385	-0.0192	0.0035	4.49E-08	0.0126	0.0118	0.2835
rs10400776	14	97326366	A	C	0.2593	-0.0259	0.0044	3.45E-09	0.002	0.0133	0.8809
rs10427614	22	29928932	A	G	0.7708	-0.0249	0.0046	4.94E-08	-0.0214	0.0139	0.1224
rs10432338	2	147806785	T	G	0.1823	0.0331	0.005	2.61E-11	0.0235	0.0151	0.1203

rs10792966	11	71544105	C	G	0.6876	0.0237	0.0041	1.06E-08	0.0204	0.0126	0.1052
rs11066368	12	109790342	A	G	0.3874	0.021	0.0036	4.31E-09	0.0046	0.012	0.704399
rs11074655	16	10300931	T	C	0.5879	-0.0205	0.0035	6.51E-09	0.0039	0.0118	0.744201
rs113367286	7	140144414	T	C	0.2787	-0.0251	0.0043	4.99E-09	-0.0057	0.013	0.6598
rs11597584	10	66401600	T	C	0.3432	-0.0224	0.0041	4.15E-08	-1.00E-04	0.0124	0.9905
rs117737827	15	63804248	T	C	0.1558	-0.0299	0.0053	1.69E-08	-0.0259	0.0161	0.1076
rs1188840	6	139214819	A	G	0.6509	0.0236	0.004	4.40E-09	-0.0153	0.0122	0.2112
rs12188899	5	59670706	A	G	0.797	-0.0274	0.0048	1.09E-08	0.0125	0.0146	0.3916
rs1219798	10	125400180	T	C	0.1746	0.0269	0.0046	4.64E-09	-0.0259	0.0154	0.092221
rs12206846	6	108238917	A	G	0.4054	0.0199	0.0036	2.10E-08	0.0027	0.0119	0.818
rs12214364	6	67556372	T	G	0.5869	-0.0197	0.0036	4.87E-08	-0.0021	0.012	0.8611
rs12469397	2	193762132	A	G	0.4922	-0.0258	0.0038	2.07E-11	-0.0149	0.0117	0.2015

rs12531466	7	98933552	T	C	0.8738	0.034	0.0061	1.91E-08	-0.0058	0.0175	0.7415
rs12707100	7	133274376	T	C	0.7984	-0.0265	0.0048	2.84E-08	0.0045	0.0145	0.7556
rs127204	1	112277693	A	C	0.5998	-0.0258	0.0039	3.59E-11	-0.0119	0.0119	0.3172
rs12992995	2	175197545	A	C	0.2755	-0.0258	0.0043	2.10E-09	-1.00E-04	0.0131	0.9969
rs13011181	2	100732446	T	C	0.7615	0.0278	0.0045	7.34E-10	-0.0138	0.0137	0.3142
rs13089152	3	84765574	T	C	0.6691	-0.0234	0.0041	1.53E-08	0.0018	0.0126	0.8873
rs13144223	4	17872707	T	C	0.8683	-0.0293	0.0051	1.19E-08	-0.0116	0.0172	0.4996
rs13168806	5	120071604	A	C	0.3752	0.0225	0.004	1.52E-08	-0.0031	0.012	0.7984
rs138225564	3	83063804	A	G	0.6479	0.0251	0.0046	4.48E-08	0.0099	0.0144	0.4907
rs1391954	11	88575965	T	G	0.4439	0.0245	0.004	1.51E-09	-0.013	0.0117	0.2686
rs144519232	3	48866313	C	G	0.9112	-0.0404	0.0068	3.00E-09	-0.021	0.0206	0.3103
rs1483796	7	49871114	A	G	0.3988	-0.0199	0.0036	2.15E-08	-0.0043	0.0119	0.7175

rs151431	4	103128298	A	G	0.2364	0.0277	0.0048	7.14E-09	0.0326	0.015	0.0296203
rs17801257	20	58892520	A	G	0.1247	-0.0292	0.0053	2.92E-08	0.0059	0.0178	0.7422
rs1837687	2	191238799	C	G	0.609	0.0222	0.0039	1.85E-08	0.0075	0.012	0.5295
rs1938450	1	68359482	A	G	0.3312	-0.0226	0.0041	2.66E-08	-0.0085	0.0124	0.4925
rs1968557	5	112029044	T	C	0.4961	-0.0193	0.0035	3.92E-08	-0.017	0.0117	0.1461
rs2344118	8	30788634	A	C	0.3611	0.0208	0.0037	2.65E-08	-0.0021	0.0124	0.8651
rs2417261	12	13414139	T	G	0.1085	0.0328	0.0056	5.88E-09	0.0099	0.0189	0.6011
rs243077	2	60616682	A	G	0.4197	0.0242	0.0039	5.26E-10	-0.0172	0.0118	0.146
rs2452681	11	65814842	A	G	0.7595	-0.0289	0.0045	1.41E-10	0.0104	0.0137	0.4465
rs2473977	6	113454213	A	T	0.3862	0.0195	0.0036	4.60E-08	0.0102	0.012	0.3946
rs27694	5	96164109	A	T	0.2087	-0.0236	0.0043	3.66E-08	-0.0328	0.0144	0.0226898
rs28808010	9	22492584	T	C	0.949	-0.0488	0.0089	4.41E-08	0.0038	0.0275	0.8889

rs2965216	19	13071082	A	G	0.4549	0.0212	0.0039	4.61E-08	-0.0134	0.0118	0.2563
rs34468927	19	37400719	A	G	0.8552	0.0301	0.0055	4.00E-08	0.0101	0.018	0.574101
rs34517439	1	78450517	A	C	0.1232	0.0368	0.0059	4.36E-10	-0.007	0.0179	0.6955
rs34898652	1	43974091	A	G	0.3825	-0.0228	0.0039	6.54E-09	-0.0337	0.012	0.00502597
rs349368	8	73427898	T	C	0.081	0.0349	0.0064	4.73E-08	0.0176	0.0215	0.4115
rs35041900	1	171451621	T	C	0.0932	0.0389	0.0066	3.77E-09	0.0122	0.0201	0.5454
rs362307	4	3241845	T	C	0.0739	0.0417	0.0075	2.64E-08	-0.0232	0.0222	0.2942
rs3781412	10	126715154	A	G	0.6005	-0.0208	0.0036	8.38E-09	-0.0122	0.0119	0.3036
rs3853705	2	44261892	T	C	0.6858	0.0228	0.004	1.43E-08	0.0066	0.0126	0.6016
rs4275659	12	123447928	T	C	0.2838	-0.0217	0.0039	1.99E-08	0.0036	0.013	0.782699
rs4311996	10	103735978	A	T	0.3873	-0.0217	0.0039	3.56E-08	0.0101	0.012	0.4007
rs4326129	5	151996816	A	T	0.6807	0.0226	0.0041	3.86E-08	-0.0032	0.0125	0.796

rs4375301	1	98298371	A	G	0.7673	-0.0265	0.0045	5.08E-09	-0.0173	0.0138	0.2113
rs4460001	4	130275243	A	C	0.4324	-0.0193	0.0035	4.45E-08	-0.0135	0.0119	0.2552
rs4599438	4	77028142	A	G	0.3867	0.0212	0.0038	3.54E-08	-0.0085	0.012	0.478
rs4615256	5	62700867	A	G	0.5275	-0.0205	0.0035	3.33E-09	-0.0176	0.0117	0.1321
rs4680609	3	165598022	A	G	0.6051	-0.0221	0.0039	1.89E-08	-0.0161	0.0129	0.2137
rs474252	11	95619292	C	G	0.3909	-0.0218	0.0038	1.29E-08	0.0133	0.0119	0.2664
rs4948288	10	63584533	A	G	0.4132	-0.0195	0.0035	3.31E-08	-0.0104	0.0118	0.3809
rs55716899	3	71498561	T	C	0.5712	0.0217	0.0039	2.24E-08	0.0075	0.0118	0.5225
rs566017137	2	157149797	C	G	0.9639	-0.0656	0.0108	1.23E-09	0.0223	0.0339	0.51
rs572994	6	12686856	T	C	0.3854	-0.0242	0.0039	8.36E-10	-0.0043	0.012	0.720299
rs58087899	1	1863026	A	G	0.4556	-0.0222	0.004	4.35E-08	-0.0241	0.0127	0.0573099
rs6010651	20	62418243	A	C	0.6201	0.0235	0.004	3.34E-09	0.0014	0.012	0.9047

rs60344242	3	70472539	T	C	0.1922	0.0271	0.0049	2.48E-08	-0.0043	0.0148	0.7732
rs6102913	20	41202958	T	C	0.4736	-0.0193	0.0035	2.97E-08	0.0038	0.0116	0.7467
rs61166637	4	140771814	C	G	0.6711	0.0242	0.0043	1.48E-08	-0.0079	0.0124	0.5213
rs61813324	1	156049877	T	C	0.1361	0.031	0.0057	4.37E-08	0.0037	0.0172	0.8287
rs62009152	15	78001011	T	C	0.7423	0.0259	0.0044	3.63E-09	0.0015	0.0133	0.9091
rs62057680	16	29986627	T	C	0.9204	-0.0408	0.0072	1.56E-08	-0.0051	0.0218	0.8155
rs62068672	16	89647015	T	C	0.798	0.0263	0.0048	4.09E-08	0.024	0.0157	0.1267
rs62129987	19	10744807	T	C	0.6259	-0.0274	0.004	5.68E-12	-0.0158	0.0131	0.2278
rs62134192	2	45053345	C	G	0.9545	0.0524	0.0093	1.68E-08	-0.0291	0.0281	0.3013
rs62275609	3	90356910	A	G	0.2509	0.0248	0.0044	2.29E-08	-0.0271	0.0146	0.0636605
rs6430085	2	146255869	T	G	0.4936	-0.0222	0.0039	8.66E-09	-0.0082	0.0117	0.484
rs657412	13	99047250	T	G	0.1047	-0.033	0.0058	1.40E-08	0.0164	0.0191	0.3906

rs66723933	5	62360995	A	C	0.1743	-0.028	0.0051	3.23E-08	-0.0091	0.0154	0.5571
rs6674314	1	243920895	A	G	0.8134	0.0274	0.0049	2.30E-08	-0.0058	0.0149	0.696
rs67369356	5	77323022	T	C	0.2451	-0.0244	0.0045	4.23E-08	-0.0158	0.0135	0.2423
rs6741157	2	104421850	T	C	0.4526	0.0223	0.0039	8.69E-09	0.012	0.0117	0.3072
rs6743939	2	215254537	T	G	0.5831	0.0203	0.0035	9.03E-09	-0.0273	0.0118	0.0209802
rs6763786	3	74966094	A	G	0.2296	0.0228	0.0041	3.30E-08	0.0018	0.0138	0.8976
rs67943625	3	50795628	A	G	0.9383	-0.0558	0.0101	3.02E-08	-0.0418	0.0316	0.1859
rs6857	19	45392254	T	C	0.1689	-0.0369	0.0047	5.80E-15	-0.0399	0.0155	0.01011
rs71639293	5	92995013	A	G	0.7974	0.0266	0.0048	2.80E-08	-0.0095	0.0145	0.5147
rs7227386	18	41594173	A	C	0.6831	-0.023	0.0041	2.48E-08	0.0097	0.0125	0.4372
rs73028181	6	166115539	T	G	0.0262	0.0704	0.012	4.40E-09	0.0482	0.0364	0.1847
rs73051647	12	6835160	A	G	0.0964	0.0377	0.0065	7.28E-09	0.0377	0.0197	0.05633

rs73420302	17	77768068	C	G	0.1785	-0.03	0.0051	3.04E-09	-0.0057	0.0153	0.7076
rs73697465	7	53900433	A	C	0.1662	0.0294	0.0052	1.39E-08	0.0266	0.0158	0.0909599
rs74996610	12	24075007	C	G	0.9545	-0.0513	0.0093	3.12E-08	-0.0098	0.0278	0.724499
rs7532726	1	209760936	A	C	0.7355	0.0218	0.0039	2.40E-08	-0.0173	0.0132	0.1906
rs7579662	2	161915810	A	G	0.393	0.0216	0.0039	4.15E-08	-0.019	0.012	0.1119
rs7803571	7	110993511	T	C	0.3722	0.0197	0.0036	4.74E-08	0.0124	0.0121	0.3025
rs78394231	6	107649123	T	C	0.9015	-0.0381	0.0065	3.53E-09	0.0436	0.0197	0.0265003
rs7843666	8	10742059	A	G	0.4938	0.0193	0.0035	3.57E-08	-0.0433	0.0117	0.000205902
rs7875078	9	14494845	A	C	0.4517	0.0204	0.0035	7.41E-09	-0.0039	0.0117	0.738901
rs9277976	6	33292745	T	C	0.9053	-0.0391	0.0069	1.41E-08	-0.0105	0.0199	0.5995
rs9411327	9	134794501	A	G	0.1654	0.03	0.0052	7.59E-09	0.0038	0.0157	0.8064
rs948537	18	35125161	T	C	0.2281	0.0277	0.0046	1.83E-09	-0.0179	0.014	0.2

rs9557370	13	100704249	A	G	0.2562	0.0301	0.0044	9.66E-12	3.00E-04	0.0134	0.9849
rs9867121	3	114631548	A	C	0.1829	-0.0317	0.005	2.02E-10	0.0032	0.0151	0.8324
rs9889540	17	60789059	C	G	0.3863	0.0234	0.0041	1.78E-08	0.005	0.012	0.680299
rs993884	5	7378895	T	C	0.6328	-0.0218	0.004	4.55E-08	-0.0049	0.0121	0.6851

Supplemental Table 3: Summary of the 108 SNPs associated with LST and overall OA. LST, leisure screen time; OA, Osteoarthritis.

SNP	chromosome	Position	Effect allele	Other allele	Effect allele frequency	Effect on LSP			Effect on overall OA		
						Beta	SE	P value	Beta	SE	P value
rs10044672	5	138290479	C	G	0.6518	-0.0233	0.0037	1.79E-10	-0.0102	0.008	0.2017
rs10159545	10	21766969	C	G	0.6504	-0.0347	0.0041	9.62E-18	-0.0259	0.008	0.00124899
rs10206568	2	217250099	C	G	0.71	0.021	0.0038	4.41E-08	0.0013	0.0084	0.878
rs10222987	4	185946130	A	G	0.6355	-0.0209	0.0036	9.02E-09	-0.0023	0.0079	0.774701
rs10273241	7	8085405	A	G	0.4385	-0.0192	0.0035	4.49E-08	-0.01	0.0077	0.1934
rs10400776	14	97326366	A	C	0.2593	-0.0259	0.0044	3.45E-09	0.0025	0.0087	0.7698
rs10427614	22	29928932	A	G	0.7708	-0.0249	0.0046	4.94E-08	0.004	0.009	0.654501
rs10432338	2	147806785	T	G	0.1823	0.0331	0.005	2.61E-11	0.0085	0.0098	0.3869
rs10792966	11	71544105	C	G	0.6876	0.0237	0.0041	1.06E-08	0.0189	0.0082	0.02121

rs11066368	12	109790342	A	G	0.3874	0.021	0.0036	4.31E-09	0.0101	0.0078	0.1956
rs11074655	16	10300931	T	C	0.5879	-0.0205	0.0035	6.51E-09	-0.0019	0.0077	0.8029
rs113367286	7	140144414	T	C	0.2787	-0.0251	0.0043	4.99E-09	-0.0089	0.0085	0.2942
rs11597584	10	66401600	T	C	0.3432	-0.0224	0.0041	4.15E-08	-0.0045	0.0081	0.579301
rs117737827	15	63804248	T	C	0.1558	-0.0299	0.0053	1.69E-08	-0.0102	0.0105	0.3297
rs1188840	6	139214819	A	G	0.6509	0.0236	0.004	4.40E-09	0.0031	0.008	0.7013
rs12188899	5	59670706	A	G	0.797	-0.0274	0.0048	1.09E-08	-0.0033	0.0095	0.7274
rs1219798	10	125400180	T	C	0.1746	0.0269	0.0046	4.64E-09	-0.0163	0.01	0.1042
rs12206846	6	108238917	A	G	0.4054	0.0199	0.0036	2.10E-08	0.0029	0.0078	0.706601
rs12214364	6	67556372	T	G	0.5869	-0.0197	0.0036	4.87E-08	-0.0055	0.0078	0.4852
rs12469397	2	193762132	A	G	0.4922	-0.0258	0.0038	2.07E-11	-0.0074	0.0076	0.331
rs12531466	7	98933552	T	C	0.8738	0.034	0.0061	1.91E-08	0.0049	0.0114	0.6707

rs12707100	7	133274376	T	C	0.7984	-0.0265	0.0048	2.84E-08	0.0086	0.0095	0.3622
rs127204	1	112277693	A	C	0.5998	-0.0258	0.0039	3.59E-11	-0.0103	0.0077	0.1845
rs12992995	2	175197545	A	C	0.2755	-0.0258	0.0043	2.10E-09	-0.0225	0.0085	0.00818993
rs13011181	2	100732446	T	C	0.7615	0.0278	0.0045	7.34E-10	0.0044	0.0089	0.6194
rs13089152	3	84765574	T	C	0.6691	-0.0234	0.0041	1.53E-08	-0.0019	0.0082	0.8154
rs13144223	4	17872707	T	C	0.8683	-0.0293	0.0051	1.19E-08	-0.0178	0.0112	0.1132
rs13168806	5	120071604	A	C	0.3752	0.0225	0.004	1.52E-08	0.0104	0.0079	0.1857
rs138225564	3	83063804	A	G	0.6479	0.0251	0.0046	4.48E-08	0.012	0.0092	0.1915
rs1391954	11	88575965	T	G	0.4439	0.0245	0.004	1.51E-09	0.0034	0.0076	0.6598
rs144519232	3	48866313	C	G	0.9112	-0.0404	0.0068	3.00E-09	-0.0334	0.0134	0.01287
rs1483796	7	49871114	A	G	0.3988	-0.0199	0.0036	2.15E-08	6.00E-04	0.0078	0.937
rs151431	4	103128298	A	G	0.2364	0.0277	0.0048	7.14E-09	0.0429	0.0096	7.24E-06

rs17801257	20	58892520	A	G	0.1247	-0.0292	0.0053	2.92E-08	0.0053	0.0116	0.6487
rs1837687	2	191238799	C	G	0.609	0.0222	0.0039	1.85E-08	0.0054	0.0078	0.4895
rs1938450	1	68359482	A	G	0.3312	-0.0226	0.0041	2.66E-08	-0.023	0.0081	0.004339
rs1968557	5	112029044	T	C	0.4961	-0.0193	0.0035	3.92E-08	-0.0262	0.0076	0.000577006
rs2344118	8	30788634	A	C	0.3611	0.0208	0.0037	2.65E-08	0.0088	0.0081	0.2775
rs2417261	12	13414139	T	G	0.1085	0.0328	0.0056	5.88E-09	0.017	0.0123	0.1669
rs243077	2	60616682	A	G	0.4197	0.0242	0.0039	5.26E-10	-0.0079	0.0077	0.3085
rs2452681	11	65814842	A	G	0.7595	-0.0289	0.0045	1.41E-10	-0.0066	0.0089	0.4608
rs2473977	6	113454213	A	T	0.3862	0.0195	0.0036	4.60E-08	-0.003	0.0078	0.705601
rs27694	5	96164109	A	T	0.2087	-0.0236	0.0043	3.66E-08	-0.0173	0.0094	0.0645907
rs28808010	9	22492584	T	C	0.949	-0.0488	0.0089	4.41E-08	1.00E-04	0.0179	0.9957
rs2965216	19	13071082	A	G	0.4549	0.0212	0.0039	4.61E-08	-0.0131	0.0077	0.088781

rs34468927	19	37400719	A	G	0.8552	0.0301	0.0055	4.00E-08	0.0098	0.0115	0.3929
rs34517439	1	78450517	A	C	0.1232	0.0368	0.0059	4.36E-10	0.0248	0.0117	0.0336001
rs34898652	1	43974091	A	G	0.3825	-0.0228	0.0039	6.54E-09	-0.0154	0.0078	0.0495097
rs349368	8	73427898	T	C	0.081	0.0349	0.0064	4.73E-08	0.002	0.014	0.8864
rs35041900	1	171451621	T	C	0.0932	0.0389	0.0066	3.77E-09	0.0054	0.0131	0.6822
rs362307	4	3241845	T	C	0.0739	0.0417	0.0075	2.64E-08	0.0149	0.0145	0.304
rs3781412	10	126715154	A	G	0.6005	-0.0208	0.0036	8.38E-09	-0.0099	0.0078	0.2002
rs3853705	2	44261892	T	C	0.6858	0.0228	0.004	1.43E-08	0.004	0.0082	0.6264
rs4275659	12	123447928	T	C	0.2838	-0.0217	0.0039	1.99E-08	-0.0294	0.0085	0.000520595
rs4311996	10	103735978	A	T	0.3873	-0.0217	0.0039	3.56E-08	0.0084	0.0078	0.2839
rs4326129	5	151996816	A	T	0.6807	0.0226	0.0041	3.86E-08	-0.0048	0.0082	0.5569
rs4375301	1	98298371	A	G	0.7673	-0.0265	0.0045	5.08E-09	-0.0197	0.009	0.0290703

rs4460001	4	130275243	A	C	0.4324	-0.0193	0.0035	4.45E-08	-0.0079	0.0077	0.3079
rs4599438	4	77028142	A	G	0.3867	0.0212	0.0038	3.54E-08	-0.0102	0.0078	0.1908
rs4615256	5	62700867	A	G	0.5275	-0.0205	0.0035	3.33E-09	-0.0182	0.0076	0.0168601
rs4680609	3	165598022	A	G	0.6051	-0.0221	0.0039	1.89E-08	-0.0139	0.0082	0.092081
rs474252	11	95619292	C	G	0.3909	-0.0218	0.0038	1.29E-08	-0.0085	0.0078	0.2757
rs4948288	10	63584533	A	G	0.4132	-0.0195	0.0035	3.31E-08	-0.0039	0.0077	0.6167
rs55716899	3	71498561	T	C	0.5712	0.0217	0.0039	2.24E-08	0.0114	0.0077	0.1374
rs566017137	2	157149797	C	G	0.9639	-0.0656	0.0108	1.23E-09	-0.0161	0.0216	0.4571
rs572994	6	12686856	T	C	0.3854	-0.0242	0.0039	8.36E-10	-0.0126	0.0078	0.106
rs58087899	1	1863026	A	G	0.4556	-0.0222	0.004	4.35E-08	-0.0161	0.0081	0.0461604
rs6010651	20	62418243	A	C	0.6201	0.0235	0.004	3.34E-09	0.0068	0.0079	0.3851
rs60344242	3	70472539	T	C	0.1922	0.0271	0.0049	2.48E-08	0.0152	0.0097	0.1145

rs6102913	20	41202958	T	C	0.4736	-0.0193	0.0035	2.97E-08	-0.0126	0.0076	0.0964606
rs61166637	4	140771814	C	G	0.6711	0.0242	0.0043	1.48E-08	0.0059	0.0081	0.4623
rs61813324	1	156049877	T	C	0.1361	0.031	0.0057	4.37E-08	0.0149	0.0112	0.1855
rs62009152	15	78001011	T	C	0.7423	0.0259	0.0044	3.63E-09	0.0079	0.0087	0.3629
rs62057680	16	29986627	T	C	0.9204	-0.0408	0.0072	1.56E-08	-0.0124	0.0142	0.3817
rs62068672	16	89647015	T	C	0.798	0.0263	0.0048	4.09E-08	0.0274	0.01	0.00634805
rs62129987	19	10744807	T	C	0.6259	-0.0274	0.004	5.68E-12	-0.0326	0.0083	8.97E-05
rs62134192	2	45053345	C	G	0.9545	0.0524	0.0093	1.68E-08	-0.0134	0.0183	0.466
rs62275609	3	90356910	A	G	0.2509	0.0248	0.0044	2.29E-08	-0.0016	0.0093	0.8656
rs6430085	2	146255869	T	G	0.4936	-0.0222	0.0039	8.66E-09	-0.0082	0.0076	0.2818
rs657412	13	99047250	T	G	0.1047	-0.033	0.0058	1.40E-08	0.0124	0.0124	0.3193
rs66723933	5	62360995	A	C	0.1743	-0.028	0.0051	3.23E-08	-0.0038	0.0101	0.7079

rs6674314	1	243920895	A	G	0.8134	0.0274	0.0049	2.30E-08	0.0076	0.0098	0.4349
rs67369356	5	77323022	T	C	0.2451	-0.0244	0.0045	4.23E-08	-0.0134	0.0088	0.1286
rs6741157	2	104421850	T	C	0.4526	0.0223	0.0039	8.69E-09	0.0225	0.0077	0.00334603
rs6743939	2	215254537	T	G	0.5831	0.0203	0.0035	9.03E-09	-0.0094	0.0077	0.2222
rs6763786	3	74966094	A	G	0.2296	0.0228	0.0041	3.30E-08	0.0116	0.009	0.1995
rs67943625	3	50795628	A	G	0.9383	-0.0558	0.0101	3.02E-08	-0.0131	0.0202	0.516
rs6857	19	45392254	T	C	0.1689	-0.0369	0.0047	5.80E-15	-0.0285	0.0101	0.00482903
rs71639293	5	92995013	A	G	0.7974	0.0266	0.0048	2.80E-08	-0.005	0.0095	0.5962
rs7227386	18	41594173	A	C	0.6831	-0.023	0.0041	2.48E-08	-0.0056	0.0082	0.4938
rs73028181	6	166115539	T	G	0.0262	0.0704	0.012	4.40E-09	-0.0214	0.0237	0.3679
rs73051647	12	6835160	A	G	0.0964	0.0377	0.0065	7.28E-09	-2.00E-04	0.0129	0.9865
rs73420302	17	77768068	C	G	0.1785	-0.03	0.0051	3.04E-09	-0.0079	0.01	0.431

rs73697465	7	53900433	A	C	0.1662	0.0294	0.0052	1.39E-08	0.0143	0.0103	0.1645
rs74996610	12	24075007	C	G	0.9545	-0.0513	0.0093	3.12E-08	-0.003	0.0182	0.8699
rs7532726	1	209760936	A	C	0.7355	0.0218	0.0039	2.40E-08	-0.0157	0.0086	0.0676099
rs7579662	2	161915810	A	G	0.393	0.0216	0.0039	4.15E-08	-0.0018	0.0078	0.8205
rs7803571	7	110993511	T	C	0.3722	0.0197	0.0036	4.74E-08	0.0193	0.0079	0.0142001
rs78394231	6	107649123	T	C	0.9015	-0.0381	0.0065	3.53E-09	0.0285	0.0128	0.02625
rs7843666	8	10742059	A	G	0.4938	0.0193	0.0035	3.57E-08	-0.0329	0.0076	1.50E-05
rs7875078	9	14494845	A	C	0.4517	0.0204	0.0035	7.41E-09	-0.0032	0.0077	0.679201
rs9277976	6	33292745	T	C	0.9053	-0.0391	0.0069	1.41E-08	-0.0116	0.013	0.3722
rs9411327	9	134794501	A	G	0.1654	0.03	0.0052	7.59E-09	0.0035	0.0102	0.7334
rs948537	18	35125161	T	C	0.2281	0.0277	0.0046	1.83E-09	-0.0016	0.0091	0.8635
rs9557370	13	100704249	A	G	0.2562	0.0301	0.0044	9.66E-12	0.0118	0.0088	0.1793

rs9867121	3	114631548	A	C	0.1829	-0.0317	0.005	2.02E-10	0.0069	0.0099	0.4828
rs9889540	17	60789059	C	G	0.3863	0.0234	0.0041	1.78E-08	4.00E-04	0.0078	0.9618
rs993884	5	7378895	T	C	0.6328	-0.0218	0.004	4.55E-08	0.008	0.0079	0.31

Supplemental Table 4: Summary of the 15 SNPs associated with MVPA and knee OA. MVPA, moderate-to-vigorous physical activity; OA, Osteoarthritis.

SNP	chromosome	Position	Effect allele	Other allele	Effect allele frequency	Effect on MVPA			Effect on knee OA		
						Beta	SE	P value	Beta	SE	P value
rs117467952	11	65854553	T	C	0.0904	0.039	0.0071	4.69E-08	-0.018	0.0163	0.2705
rs12357890	10	99762693	A	G	0.4431	0.0225	0.0041	4.77E-08	-0.0102	0.0099	0.3035
rs12939066	17	50270268	T	C	0.3187	-0.0188	0.0034	3.27E-08	0.0285	0.0098	0.003804
rs13085795	3	84857121	A	G	0.7518	-0.0202	0.0037	4.35E-08	-0.0064	0.0105	0.5412
rs166840	17	19799698	A	G	0.4118	-0.0241	0.0042	7.35E-09	-0.0217	0.0095	0.0224399
rs2309757	2	100807725	T	C	0.5998	-0.0241	0.0041	6.03E-09	0.0231	0.0095	0.0149899
rs2668196	3	165502709	A	T	0.1908	-0.0227	0.004	2.09E-08	0.0292	0.0118	0.01344
rs334954	3	18625286	A	G	0.3033	0.0241	0.0044	4.85E-08	-0.0133	0.0101	0.1869

rs4352559	5	60586625	T	C	0.4964	0.018	0.0032	1.65E-08	-0.0049	0.0093	0.6011
rs4656648	1	169092123	T	C	0.537	0.0226	0.0041	2.69E-08	0.0013	0.0093	0.8865
rs4865512	5	50661601	A	G	0.6124	0.024	0.0042	7.68E-09	0.0057	0.0095	0.5479
rs488769	11	57450720	A	C	0.6503	0.0183	0.0033	3.25E-08	0.0049	0.0098	0.6194
rs568546	11	107321156	T	C	0.5206	0.0237	0.0041	5.89E-09	-0.0105	0.0093	0.2588
rs6924664	6	141790263	T	G	0.2653	-0.0201	0.0036	2.45E-08	-0.0074	0.0105	0.484
rs6925193	6	26475016	T	C	0.2118	0.0275	0.005	3.05E-08	-0.0423	0.0114	0.000197802

Supplemental Table 5: Summary of the 15 SNPs associated with MVPA and hip OA. MVPA, moderate-to-vigorous physical activity; OA, Osteoarthritis.

SNP	chromosome	Position	Effect allele	Other allele	Effect allele frequency	Effect on MVPA			Effect on hip OA		
						Beta	SE	P value	Beta	SE	P value
rs117467952	11	65854553	T	C	0.0904	0.039	0.0071	4.69E-08	0.0489	0.0204	0.0166702
rs12357890	10	99762693	A	G	0.4431	0.0225	0.0041	4.77E-08	0.0077	0.0127	0.5437
rs12939066	17	50270268	T	C	0.3187	-0.0188	0.0034	3.27E-08	-0.0078	0.0124	0.5282
rs13085795	3	84857121	A	G	0.7518	-0.0202	0.0037	4.35E-08	-0.0112	0.0132	0.3966
rs166840	17	19799698	A	G	0.4118	-0.0241	0.0042	7.35E-09	0.0046	0.0119	0.696599
rs2309757	2	100807725	T	C	0.5998	-0.0241	0.0041	6.03E-09	-0.0092	0.0119	0.4405
rs2668196	3	165502709	A	T	0.1908	-0.0227	0.004	2.09E-08	0.0285	0.0148	0.0546298
rs334954	3	18625286	A	G	0.3033	0.0241	0.0044	4.85E-08	0.0232	0.0127	0.0676597

rs4352559	5	60586625	T	C	0.4964	0.018	0.0032	1.65E-08	0.0151	0.0117	0.1959
rs4656648	1	169092123	T	C	0.537	0.0226	0.0041	2.69E-08	-0.0064	0.0117	0.5844
rs4865512	5	50661601	A	G	0.6124	0.024	0.0042	7.68E-09	0.0022	0.012	0.8514
rs488769	11	57450720	A	C	0.6503	0.0183	0.0033	3.25E-08	-0.0293	0.0123	0.0174201
rs568546	11	107321156	T	C	0.5206	0.0237	0.0041	5.89E-09	0.0135	0.0117	0.249
rs6924664	6	141790263	T	G	0.2653	-0.0201	0.0036	2.45E-08	-0.0227	0.0132	0.08682
rs6925193	6	26475016	T	C	0.2118	0.0275	0.005	3.05E-08	-0.0263	0.0142	0.0650804

Supplemental Table 6: Summary of the 15 SNPs associated with MVPA and overall OA. MVPA, moderate-to-vigorous physical activity; OA, Osteoarthritis.

SNP	chromosome	Position	Effect allele	Other allele	Effect allele frequency	Effect on MVPA			Effect on overall OA		
						Beta	SE	P value	Beta	SE	P value
rs11130222	3	49901060	A	T	0.5733	0.0233	0.0041	1.39E-08	-0.0271	0.0077	0.000414696
rs117467952	11	65854553	T	C	0.0904	0.039	0.0071	4.69E-08	0.0095	0.0133	0.4767
rs12357890	10	99762693	A	G	0.4431	0.0225	0.0041	4.77E-08	-0.0051	0.0081	0.5331
rs12939066	17	50270268	T	C	0.3187	-0.0188	0.0034	3.27E-08	0.0149	0.0081	0.0644496
rs13085795	3	84857121	A	G	0.7518	-0.0202	0.0037	4.35E-08	-0.0124	0.0086	0.1488
rs166840	17	19799698	A	G	0.4118	-0.0241	0.0042	7.35E-09	-0.0119	0.0078	0.1243
rs2309757	2	100807725	T	C	0.5998	-0.0241	0.0041	6.03E-09	0.0104	0.0078	0.1795
rs2668196	3	165502709	A	T	0.1908	-0.0227	0.004	2.09E-08	0.0286	0.0097	0.00308099

rs334954	3	18625286	A	G	0.3033	0.0241	0.0044	4.85E-08	0.0028	0.0083	0.730999
rs4352559	5	60586625	T	C	0.4964	0.018	0.0032	1.65E-08	0.0015	0.0076	0.8491
rs4656648	1	169092123	T	C	0.537	0.0226	0.0041	2.69E-08	-7.00E-04	0.0076	0.9309
rs4865512	5	50661601	A	G	0.6124	0.024	0.0042	7.68E-09	0.0047	0.0078	0.5499
rs488769	11	57450720	A	C	0.6503	0.0183	0.0033	3.25E-08	-0.0045	0.008	0.5762
rs568546	11	107321156	T	C	0.5206	0.0237	0.0041	5.89E-09	-5.00E-04	0.0076	0.9529
rs6924664	6	141790263	T	G	0.2653	-0.0201	0.0036	2.45E-08	-0.0152	0.0086	0.0781502
rs6925193	6	26475016	T	C	0.2118	0.0275	0.005	3.05E-08	-0.0359	0.0093	0.000114201
